# Psychometric assessment of patients with treatment-resistant schizophrenia

**DOI:** 10.1192/j.eurpsy.2024.1560

**Published:** 2024-08-27

**Authors:** V. Kaleda, D. Tikhonov

**Affiliations:** Mental Health Research Centre, Moscow, Russian Federation

## Abstract

**Introduction:**

Treatment-resistant schizophrenia (TRS) is one of the most pressing issues in the field of treatment and research of psychotic disorders. The pronounced decline in social and professional functioning in this group of patients as well as high costs of therapy determine high interest in TRS. This is a part of an ongoing study on the clinical and biological features of TRS.

**Objectives:**

The aim of this study is to identify the leading symptoms in patients with TRS.

**Methods:**

Using the Personal and Social Performance Scale (PSP), Positive and Negative Syndrome Scale (PANSS) and Calgary Depression Scale for schizophrenia (CDSS), 30 male patients (age 28.99 ± 8.08 years) diagnosed with paranoid schizophrenia (F20.0) were examined. All patients had persistent productive symptoms and met the criteria for TRS. The average daily doses of antipsychotics in chlorpromazine equivalent were 1382.07 ± 897.15 mg/day. The average age of onset of the disease was 19.52 ± 5.97 years, the average disease was 9.47 ± 7.61 years.

**Results:**

The average scores were: on the PSP scale: 46.05 ± 9.17, on the CDSS scale 8.10 ± 4.53, on the PANSS positive symptoms subscale - 21.52 ± 4.24, on the PANSS negative symptoms subscale - 24.67 ± 4.42, on the general psychopathology subscale PANSS – 45.62 ± 6.11 . Positive symptoms were represented mainly by delusions (P1, 4.14 ± 0.85 points) and hallucinations (P3, 4.10 ± 1.76 points). Blunted affect (N1, 4.29 ± 0.56 points) and emotional withdrawal (N2, 3.67 ± 0.73 points) predominated among negative symptoms, while the least prominent negative symptom was poor rapport (N3, 3.24 ± 0.94). The most pronounced general psychopathology symptoms were depression (G6, 4.00 ± 1.10 ) and lack of judgment and insight (G12, 4.05 ± 0.92). The total score on the PANSS was 91.81 ± 12.40.

**Conclusions:**

The CDSS score indicates a high incidence of depressive symptoms in patients with TRS. A low PSP score reflects poor social functioning. The most common symptoms according to the PANSS are delusions, hallucinations, blunted affect, emotional withdrawal, depression and lack of judgment and insight.

**Disclosure of Interest:**

None Declared

